# Spinal malformations in a naturally isolated Neotropical fish population

**DOI:** 10.7717/peerj.12239

**Published:** 2021-10-20

**Authors:** Franciele Fernanda Kerniske, Jonathan Pena Castro, Luz Elena De la Ossa-Guerra, Bruna Angelina Mayer, Vinícius Abilhoa, Igor de Paiva Affonso, Roberto Ferreira Artoni

**Affiliations:** 1 Graduate Program in Evolutionary Biology, State University of Ponta Grossa, Ponta Grossa, Paraná, Brazil; 2 Graduate Program in Evolutionary Genetics and Molecular Biology, Federal University of São Carlos, São Carlos, São Paulo, Brazil; 3 Capão da Imbuia Natural History Museum, Curitiba, Paraná, Brazil; 4 Federal Technological University of Paraná, Ponta Grossa, Paraná, Brazil

**Keywords:** Inbreeding, Spinal, Ontogenetic development, Gene flow

## Abstract

Fish populations that reside in completely isolated freshwater ecosystems are rare worldwide. The Vila Velha State Park (VVSP), located in southern Brazil, is recognized for its arenitic formations called sinkholes (*furnas*), which are completely isolated. Fish populations within, such as those of *Psalidodon* aff. *fasciatus*, often develop vertebral malformations due to this isolation from other conspecifics and other species. In this study, we analyzed geometric morphology in digital radiographs to identify congenital deformations of *Psalidodon* aff. *fasciatus* in *Furna 2* of VVSP. We found many fish with spinal deformities, including wide variation in the number of caudal vertebrae and corporal deformations related to a flattened body and spinal curvature. Females were more affected than males. We also demonstrated that these deformations reflect inbreeding and an absence of gene flow in the population. In conclusion, isolated populations such as fish species in *furnas* are potential models for evo-devo research.

## Introduction

Fish species may develop different types of morphological deformities, such as dysplasia in the opercular bones, maxillary-mandibular apparatus, and mandibular bones; anomalies in fins and eyes (Mise, Tencatt & Santos, 2017); bone and skin neoplasms; and spine deformations (Flores-Lopes, Cetra & Malabarba, 2010). Dorsoventral (lordosis), lateral (scoliosis) or upward spinal curvature (kyphosis) malformations can cause anomalous body appearance (Bengtsson, Bengtsson & Himberg, 1985) and be treated as monstrosities (Golubtsov, Korostelev & Levin, 2021), but such anomalies are often not visible if only a few vertebrae are affected (Gjerde, Pante & Baeverfjord, 2005; Kvellestad et al., 2000; Witten et al., 2006). Body and skeletal malformations have been reviewed in many publications and they are related to multiple physiological, environmental, xenobiotic, nutritional and genetic factors (Slooff, 1982; Wells & Cowan, 1982; Toften, 1996; Gjerde, Pante & Baeverfjord, 2005; Jagiełło et al., 2017; Madsen, Arnbjerg & Dalsgaard, 2001). Environmental factors have been linked to vertebral anomalies in *Salmo solar* (Fraser et al., 2015; Ytteborg et al., 2010) and *Solea senegalensis* (Pimentel et al., 2014), whereas chemical substances were responsible for craniofacial deformities in *Sebastiscus marmoratus* (Zhang et al., 2012) and *Danio rerio* (Baker, Peterson & Heideman, 2013), and nutritional conditions covered a variety of deformities reported in finfish farming (Berillis, 2015; Eissa et al., 2021). Although genetic factors are usually considered marginal (reviewed in Boglione et al., 2013), alteration in extracellular matrix gene transcription in Atlantic salmon (Ytteborg et al., 2010), congenital ocular malformation and skeletal abnormalities in zebrafish (Babcock et al., 2014), deformities in bream (Afonso et al., 2000; Afonso et al., 2009) and intrinsic correlation between lordosis and consanguinity (Izquierdo, Socorro & Roo, 2010) are described in the literature. Skeletal deformities seem to be related to the early stages of development (Longwell et al., 1992), however we still know little about the genetic factors responsible for such deformations (Gjerde, Pante & Baeverfjord, 2005; Jagiełło et al., 2017; Kincaid, 1976).

Although morphological anomalies can occur both in farmed and natural fish populations, they are better investigated in aquaculture because deformities can downgrade fish production and result in economic losses (Kause et al., 2005). Culture systems usually lead to a loss of genetic variability in stocks, causing inbreeding depression and associated deformities (Aulstad & Kittelsen, 1971). Inbred populations are more susceptible to environmentally induced mortality, while also experiencing hampered growth rates and reproduction. Inbreeding can also impose difficulties for natural populations because it reduces intrapopulational genetic variability (Kause et al., 2005) and increases frequency of deleterious alleles that are normally expressed in recessive homozygotes (Keller, 2002). Even considering that the cost of inbreeding remains largely unknown and controversial (Pusey & Wolf, 1996; Crnokrak & Roff, 1999; Frommen et al., 2008), consequences can increase extinction vulnerability (Aulstad & Kittelsen, 1971; Gilpin & Soulé, 1986; Keller, 2002).

The principal aim of this investigation was to describe morphological deformities in a naturally isolated characin population of *Psalidodon* aff. *fasciatus* within a conservation unit in southern Brazil. Fish are confined to a sinkhole called *Furna 2* in the Vila Velha State Park (VVSP), a doline-shaped sandstone depression that reaches the water table. This depression possesses a mean diameter of 80 m, water depth of 50 m and walls reaching up to 110 m (Campos & Dalcomune, 2011). The formation of these sinkholes occurs by the action of acid pluvial waters that due to vast network of existing fractures in sandstones had facilitated the infiltration (Letenski et al., 2011). Briefly, the geological history of *furnas* de Vila Velha begins with the formation processes of the Paraná basin, from the Upper Ordovician to the Upper Cretaceous (Assine, 1996). Some stratigraphic graphs from the Late Carboniferous to the Upper Permian were analyzed by Holz et al. (2010), to contribute to the knowledge of the Vila Velha Sandstone formation as a result of the stacking of three lobes spread around 30 m thick each and 2 km wide, probably deposited during the excavation of the incised Lapa valley. On the other hand, weathering processes in Vila Velha responsible for the pseudo-karstic characteristics started 35 My ago, being more intense between 17 and 9 My (Riffel et al., 2015). With this, the population of *P*. aff. *fasciatus* inhabiting in the *Furna 2* can contribute to studies of spine evolutionary developmental mechanisms, supporting idiographic details as well as observed in the cavefish *Astyanax mexicanus* (Jenner & Wills, 2007) for tissue and organ studies with an evo-devo approach. Also, the geological configuration of the *furna* prevents immigration of other species, leading to population isolation and inbreeding (Artoni et al., 2006; Matoso et al., 2013; Shibatta & Artoni, 2005). The population’s endemicity, isolation, and absence of gene flow (Artoni et al., 2006; Shibatta & Artoni, 2005) makes it a useful model for our research into the effects of endogamy on morphology, since ontogenetic mechanisms can be explored and evolutionary variation can be studied under these conditions. Furthermore, this rare natural occurrence will provide an opportunity to deepen the knowledge about the functional and evolutionary biology of fish.

## Materials & methods

### Specimen sampling

**Figure 1:**
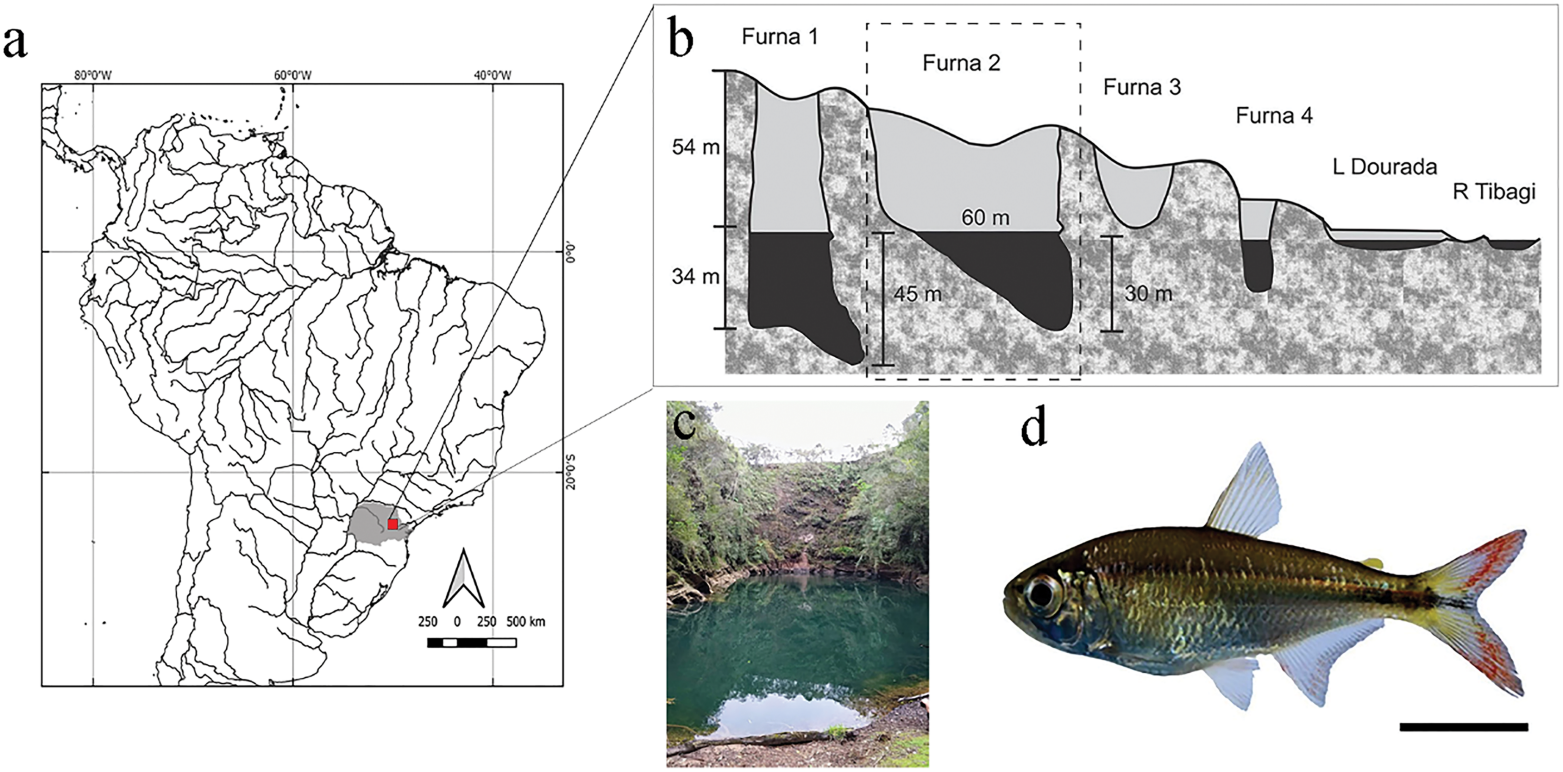
Location and layout of *furnas* in Vila Velha State Park, Paraná, Brazil. (A) Detail of South American hydrography, with emphasis on the collection site. (B) Profile of *furnas* with emphasis on *Furna 2*, also represented in (C). (D) Representative specimen of *Psalidodon* aff. *fasciatus*. Bar = 1 cm.

Specimens of *Psalidodon* aff. *fasciatus* (22 males and 13 females) were randomly collected in the sinkhole (Fig. 1) during October and November 2019, using traps and trawling of 3 m length and 5 mm mesh. Specimens were submerged in 1% benzocaine and immediately preserved in 70% alcohol. Subsequently, they were transported to the Laboratório de Ictiologia de Riberão Preto (LIRP) for radiography, then screened morphological deformations using precision scales and a digital pachymeter for total weight and standard-length measurement, respectively. Initially, fish sex was determined *via* presence of hooks on the anal fin (males), then confirmed after radiography with analysis of gonads under an optical microscope.

Research was conducted based on international guidelines of animal experimentation and authorized by the Ethical Committee in Animal Research of the Universidade Tecnológica Federal do Paraná (CEUA protocol # 2018-025/2018). Sampling was authorized by the Ministério do Meio Ambiente (MMA/ICMbio # 15115-1) and Instituto Ambiental do Paraná (IAP # 15.190.528-5; Authorization 15.18). Specimens were identified and deposited under voucher number MHNCI 13001 in the Ichtiological collection of Museu de História Natural do Capão da Imbuia, Curitiba, Paraná, Brazil.

### Radiography

Digital X-ray images of each specimen’s left side were obtained using a Faxitron (model LX-60) in the Ichthyology Laboratory of the University of São Paulo, Campus Ribeirão Preto (LIRP). Exposure time and radiation intensity were automatically calibrated for each sample (10–18 s; Kv from 26 to 31).

Total vertebral count was determined using digital radiographs (Ackerly & Ward, 2016), excluding the Weberian apparatus. In addition, standard length and total weight per individual were obtained to discriminate between sexes. According to the spine formation degree we classified the individuals as: normal pattern (Np), no deformation, the vertebral counting with approximately 32 vertebrae and no lordosis signal; moderate malformation (Mm), minimal spine deviation, individuals can present a lower vertebrae number, also, compressed or/and melted; severe malformation (Sm), high spine deviation, characterized by necessarily possess a lower number of vertebral and neural and hemal spinal anomalies, also lordotic (Kayim, Can & Guner, 2010).

Morphometric comparisons also used images of three radiographed *Psalidodon* aff. *fasciatus* specimens (LIRP Collection, voucher number 14,899) from the middle Mogi–Guaçu River, Pirassununga, São Paulo.

### Geometric morphometrics

**Figure 2:**
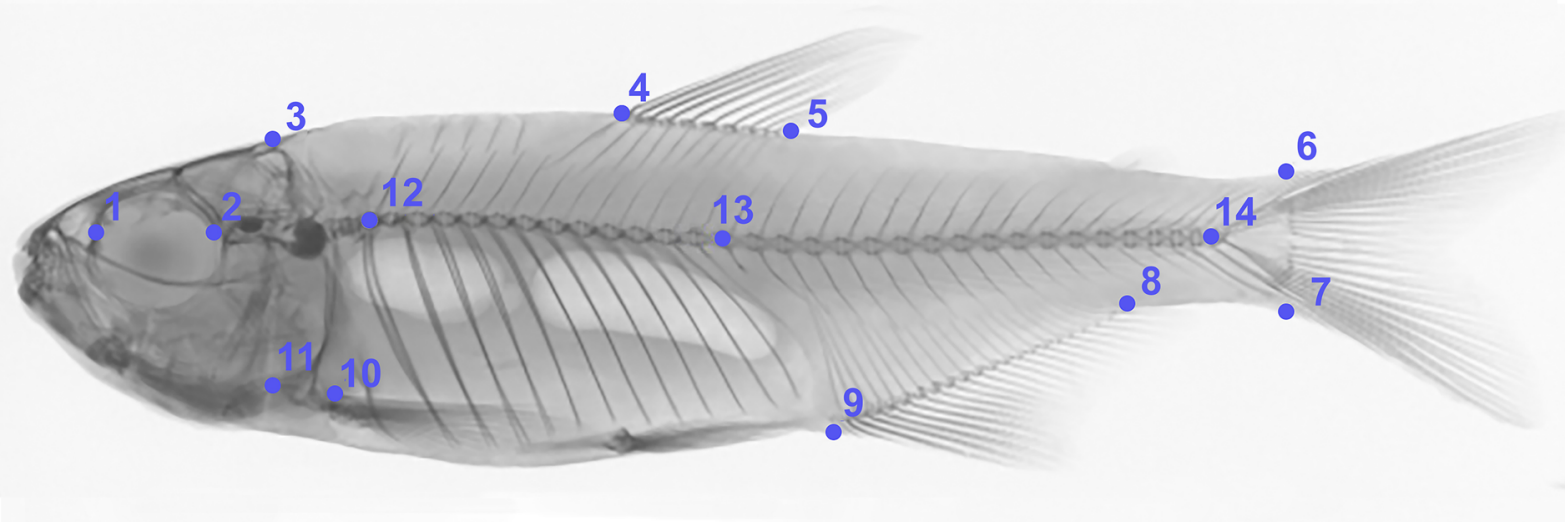
Anatomical landmarks used in X-ray images for morphometric analysis. 1: Anterior orbital region; 2: Posterior orbital region; 3: Dorsal region of head; 4: Anterior region of dorsal fin insertion; 5: Posterior region of dorsal fin insertion; 6: Dorsal region of beginning of caudal fin; 7: Ventral region of beginning of caudal fin; 8: Posterior region of anal fin insertion; 9: Anterior region of anal fin insertion; 10: Insertion of pectoral fin; 11: Ventral region of head (1–11 based on Castro et al., 2014). 12: First pre-caudal vertebra; 13: First caudal vertebra; 14: Last caudal vertebra (12–14 based on Bird & Mabee, 2003), where pre-caudal vertebrae are those posterior to the Weberian apparatus and anterior to caudal vertebrae. Pre-caudal vertebrae comprise centers, arches and neural spines, and parapophyses and ribs. Preventive and/or the first caudal vertebra can be categorized as a “transitory” vertebra, exhibiting non-fused hematoid arches or parapophyses, drastically shortened ribs, and absence of a spine. Caudal vertebrae comprise centers, neural arches and neural spines, and hematous arches and spines.

Digital radiographs were converted into a TPS file using the tpsUtil software (Rohlf, 2019). The TPS file was used to demarcate morphometric points in the TPSDig2 software (Rohlf, 2019). To produce morphometric data, we considered 14 anatomical landmarks (Fig. 2) distributed throughout the body, according to (Castro et al., 2014). For determination of errors in the landmark points, the procedure was realized in triplicate by the same observer in different moments and tested by a Procrustes ANOVA (*p* < 0.0001) performed in Morpho J v1.07a (Klingerberg, 2011).

### Statistical analysis

Procrustes superposition with minimum squares and necessary analyses such as canonical variables (CVA) and principal components (PCA) were performed in Morpho J v1.07a (Klingerberg, 2011) using the landmark means. We used three classifiers for performed the PCA and CVA: sex, locality and malformation degree. All results were statistically evaluated using the distance between groups from the distribution T-square of Hotelling and Mahalanobis (10,000 permutations; *p* < 0.0001).

## Results

### Radiographs and samples

The total number of vertebrae across all males and females ranged from 23 to 32 (Table 1; Supplementary Material 1). Number of caudal vertebrae ranged from 12 to 20, while precaudal vertebral counts were more stable and ranged from 11 to 12. Females were most variable in vertebrae count and total weight, leading to some outliers. However, females had the lowest total length.

**Table 1:** Standard length and total weight of specimens.

Individuals	Total number	Standard length (mm)	Total weight (g)
Males	22	58.92 ± 2.54	3.63 ± 0.65
Females	13	53.24 ± 4.77	3.38 ± 0.86

**Note:**

Data are displayed as means ± SD.

**Figure 3:**
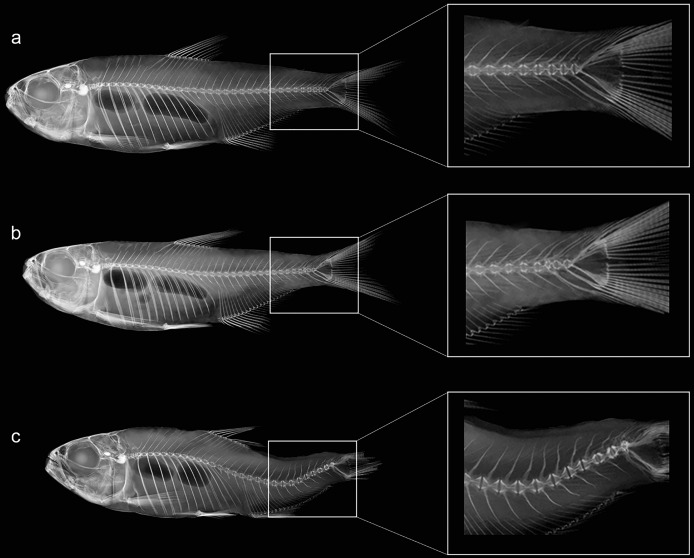
Anatomical patterns established for *Psalidodon* aff. *fasciatus* from *Furna 2* in VVSP, based on X-ray images of spine morphology. (A) Normal pattern (Np)–no deformation; (B) moderate malformation (Mm)–minimal spine deviation; (C) severe malformation (Sm)–high spine deviation.

**Figure 4:**
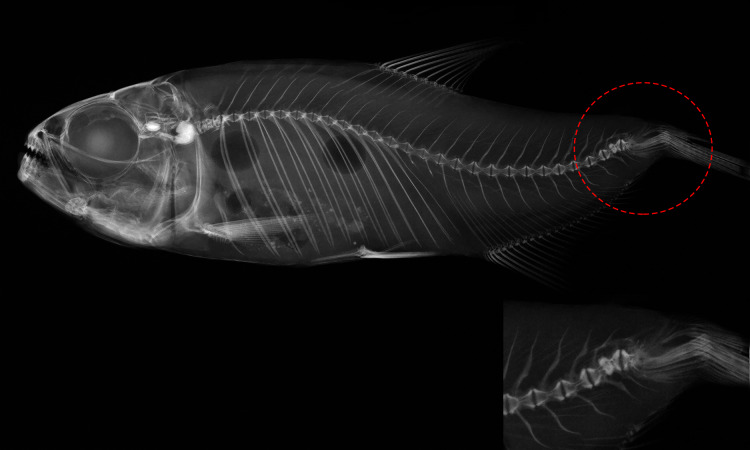
Deformed vertebral column of *Psalidodon* aff. *fasciatus* showing abnormalities in the caudal fin region, such as extra-ossifications and fusions of caudal vertebral bodies, vertebral compressions, neural and hemal spinal anomalies, ossification.

Individuals were grouped based on existing categories for deformation type (Kayim, Can & Guner, 2010): normal pattern (Np), no deformation; moderate malformation (Mm), minimal spine deviation; severe malformation (Sm), high spine deviation (Fig. 3; Table 2). In this population, females were the most affected (*p* < 0.05) comprising all individuals in the category (Sm), exhibiting downward curvature of the spine (lordosis), fused and compressed vertebrae, additionally to a reduced number of caudal vertebrae (Fig. 4). Males and females in the category (Mm), with subtle deformities corresponding to 62.5% and 37.5%, respectively. Individuals considered normal (Np) represented males in 87.5%.

**Table 2:** Chi-square test of independence between the number of males and females of *Psalidodon* aff. *fasciatus* with patterns of vertebral anomalies.

Individuals	Normal	Moderate	Severe	Total
Males	7 (87.5%)	15 (62.5%)	0	22 (62.9%)
Females	1 (12.5%)	9 (37.5%)	3 (100%)	13 (37.1%)
Total	8	24	3	35

**Note:**

The number of males and females differed significantly among the patterns of anomalies (χ^2^ = 7.159, df = 2, *P* = 0.027).

### Geometric morphometrics

**Figure 5:**
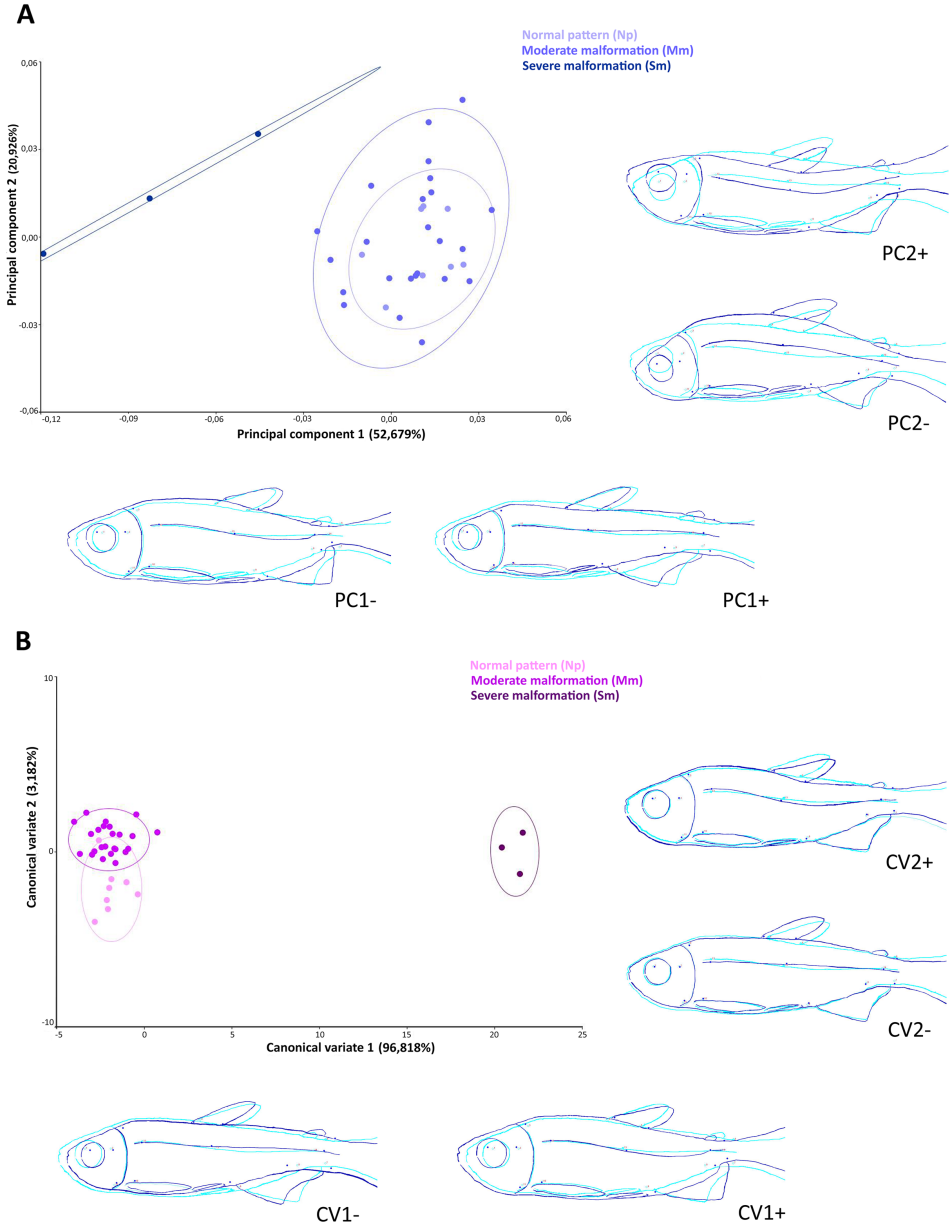
Multivariate analysis of *Psalidodon* aff. *fasciatus* of Furna 2 according to the deformities. (A) Principal components analysis (PCA). (B) Canonical variation analysis (CVA). Representation of deformations (dark blue) for each canonical variable (CV) in relation to the reference configuration (light blue) in fishes.

The scatter plot of the principal components scores on the first (52.7% of the total variation) and second (20.9% of the total variation) factorial planes (Fig. 5A), based on the 14 anatomical landmarks recorded on the 38 specimens of *Psalidodon* aff. *fasciatus*, showed an overlap of individuals classified as Normal pattern (Np) and moderate malformation (Mm), and a discrimination of individuals with severe malformation (Sm). The overlap between individuals is due to the deeper and compressed body shape, whereas individuals with severe anomalies showed a visually-detectable spinal curvature along caudal peduncle.

The Canonical variates analysis by malformation degree showed a clustering for each (Fig. 5B). The CV1 explained the 96.8% of the total variation, exhibiting individuals with severe malformations in the positive portion, with similar body shape to that identified in the negative portion of axis 1 in the PCA analysis. In both multivariate analyzes this grouping was kept completely separated. The CV2 axis explained the 3.2% of the variation, showing lordotic and normal pattern individuals in the positive and negative axis, respectively.

**Figure 6:**
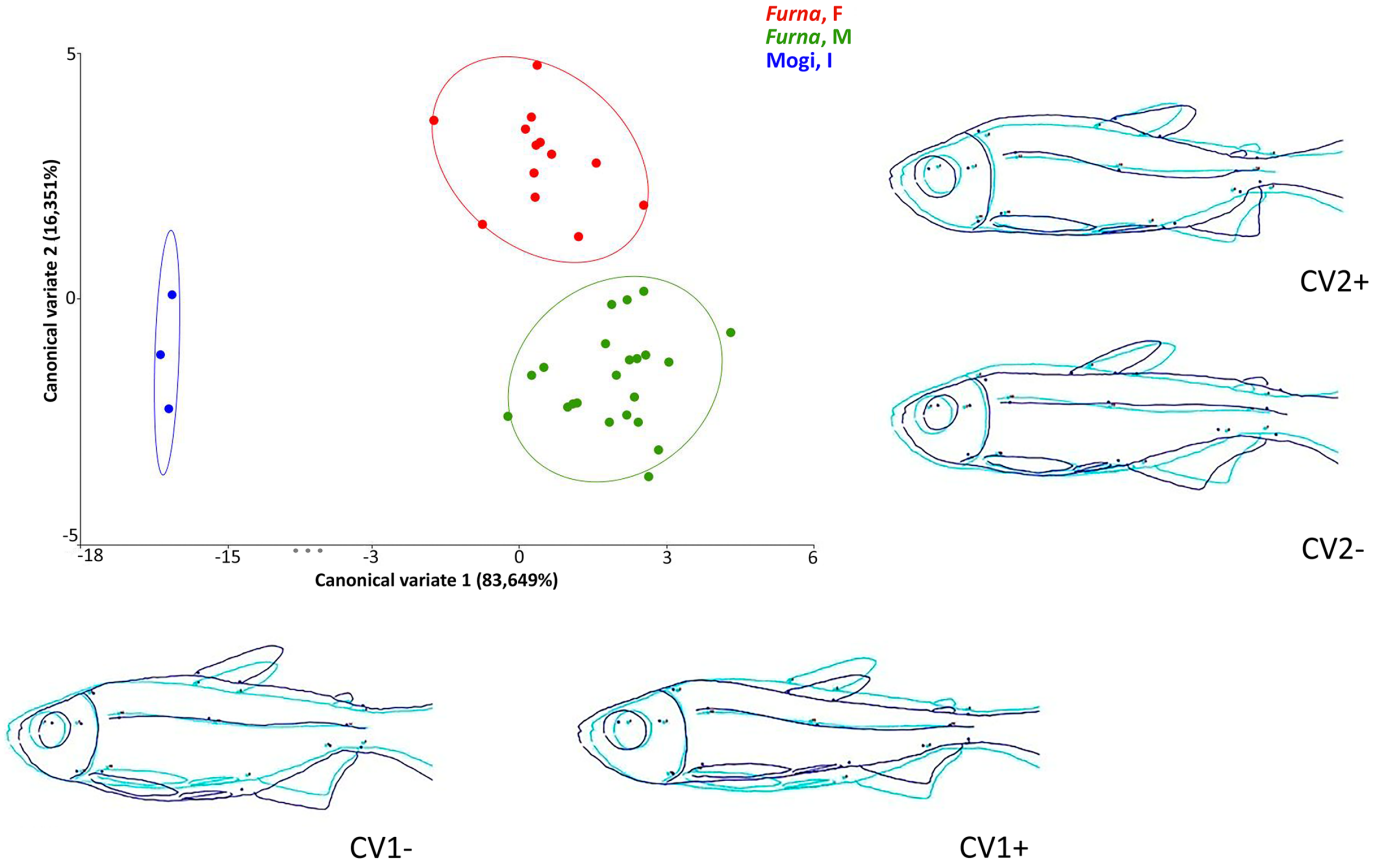
Canonical variate analysis (CVA) of *Psalidodon* aff. *fasciatus* morphology. Representation of deformations (dark blue) for each canonical variable (CV) in relation to the reference configuration (light blue) in fishes. (Furna, F) = female specimens of *Furna 2*; (Furna, M) = male specimens of *Furna 2*; (Mogi, I) = specimens from Rio Mogi-Guaçu with undetermined sex.

Multivariate analyses of canonical variates (CVA) among populations indicated morphometric differences (P < 0.0001) between Rio Mogi-Guaçu and *Furna 2* (canonical variable 1 = locality), as well as between males and females from *Furna 2* (canonical variable 2 = sex) (Fig. 6).

The first canonical axis explained 83,6% of the variation. Localities were completely separated, with *Furna 2* samples on the positive axis and Mogi-Guaçu samples on the negative axis. The former set of individuals were more elongated and compressed laterally, while the latter were more robust and broader. The second canonical axis explained 16,3% of the variation, representing a distinction between male and female individuals from *Furna 2*. Females were on the positive axis, with a higher dorsoventral length, while males on the negative axis had elongated and compressed laterally body.

## Discussion

Our results showed that individuals with spinal deformities varied widely in the number of caudal vertebrae and body deformations, notably flattened bodies and curvature in the spine. Females were the most affected in this work, however, the specific mechanisms involved are still unknown. The sample size, although small, was representative for the purpose of this study. At the same time, it is a small population that experiences conditions that can threaten its existence, which requires the utmost care so that our actions do not negatively impact it. Shortening of the spine has recently been described in natural populations of the African fish *Labeobarbus* (Cyprinidae) by Golubtsov, Korostelev & Levin (2021). These authors were unable to define possible genetic and/or environmental effects on the spinal shortening in these fish. However, they suggest that this phenotype may attribute some adaptive anti-predator advantage to the species. In contrast, our results in a completely isolated natural population of *Psalidodon* aff. *fasciatus*, suggest that spinal shortening as well as malformations are not stochastic events and can be attributed to population processes more probably associated with inbreeding. Eissa et al. (2009) associated inbreeding with lordosis, spinal fusion and other skeletal deformities in freshwater fish, also, in a study with salmonids, severely deformed individuals were more frequently homozygous (Tiira, Piironen & Primmer, 2006). Additionally, severe deformities are rarely observed in natural environments, due to these animals hardly survive, increasing the mortality of eggs and larvae (Tiira, Piironen & Primmer, 2006).

Males exhibited thinner, more elongated bodies than females, while also possessing a more stable number of vertebrae. Females exceed males in dorsoventral width and variation in vertebral number, but are lighter and shorter.

Number of vertebrae is associated with body shape, with more elongated bodies tending to exhibit more vertebrae (Ward & Brainerd, 2007). Coupled with this observation, our data suggest a likelihood of sex dimorphism. We also found morphometric differences reflecting unique corporal features in the endogamic *Furna 2* population, which was completely separated from Mogi-Guaçu individuals. However, the inbred population is not identical; Gross et al. (2004) had already called attention to the occurrence of at least two *Psalidodon* aff. *fasciatus* phenotypes in *Furna 2*, with one previously known as *Astyanax* aff. *scabripinnis*. Two possible explanations exist for these different phenotypes: natural phenotypic plasticity and genetic alterations (congenital malformations).

Variations in the vertebrae number can occur independently, because they are controlled by factors such as Hox gene expression (Buchan et al., 2014) and somitogenesis duration (Ward & Brainerd, 2007). Differences in pre-caudal and caudal vertebrae number can influence swimming (Ackerly & Ward, 2016), as the pre-caudal region contains muscles used for displacing water and caudal vertebrae support tail muscles (Reyes Corral & Aguirre, 2019). In addition, skeletal deformities can cause low weight gain, and to influence in the growth and reproduction (Eissa et al., 2021).

Thus, we can hypothesize a lower adaptive value for *Psalidodon* aff. *fasciatus* with fewer vertebrae and severe spine malformation (8.571%) than normal individuals (22.857%) or those with moderate malformation (68.571%). However, considering the absence of other fish inside *Furna 2* does not contain other fish species as competitors or predators. Combined with the abundant food availability (Mayer et al., 2021, in preparation), malformed fish still survive and reach reproductive age.

The shorter length of malformed individuals is due to the influence of lordosis on growth and an abnormal ventral curvature in the spine (Castro et al., 2008). Under other environmental conditions, excessive muscular activity of the caudal fin under strong water currents can induce lordosis (Kihara et al., 2002). However, *Furna 2* fish live in a constant lentic environment, leading us to infer that other factor (*i.e*., congenital malformations) cause lordosis. Some authors have suggested genetic associations for spinal abnormalities as observed in lordosis-scoliosis-kyphosis (LSK) syndrome (Afonso et al., 2000). In addition, besides correlation between lordosis and consanguinity, lordotic individuals exhibited fused and reduced vertebrae (Izquierdo, Socorro & Roo, 2010). Also, Afonso et al. (2009) revealed a high incidence of deformed larvae due to consanguinity and lordosis levels in seabream breeders, indicating an additive genetic effect. An induced mutation model has been established for zebrafish (*Danio rerio*), showing non-separation of vertebrae during larval development that yields different degrees of lordosis in adult fish (Gray et al., 2014). From this, evolutionary development mechanisms involved in specific traits allow to have an understanding for generalize aspects in biology (Jenner & Wills, 2007). Teleost fish represent a variable group within craniates that have demonstrated possess significantly characters suggesting being a good model (*i.e*., zebrafish). Likewise, modern evo-devo models focus on deeply conserved gene-centered mechanisms (Bolker, 2014) such as our object of study *P*. aff. *fasciatus* for the study of genetics and mechanisms related to the development of the spine. This is a promising avenue to pursue in future research on the morphology of *Psalidodon* aff. *fasciatus* from *Furna 2*.

Multiple alternative causes could explain vertebral deformations in fish, such as water temperature and turbulence (Reyes Corral & Aguirre, 2019), exposure to infectious organisms (Madsen, Arnbjerg & Dalsgaard, 2001), along with other physical, chemical, and nutritional factors (Gjerde, Pante & Baeverfjord, 2005; Madsen, Arnbjerg & Dalsgaard, 2001). Likewise, vertebral deformities, such as fusions and compressions, can occur during the embryonic period and increase throughout the life cycle (De Clercq et al., 2017). Thus, we can not discard that these factors may exert some influence on this population. On the other hand, the VVSP is a conservation unit that guarantees the preservation of natural ecosystems, while *Furna 2* in particular is very isolated, precluding regular human contact with animals (IAP, 2004). Although we do not know how long this population has been isolated, a lack of obvious external causes indicates that we should focus on genetic factors as the primary source of observed malformations. From the last report (Gross et al., 2004) to the new registries, at least 15 generations have passed, revealing the heritability and recurrence of malformed phenotypes. Thus, inbreeding and restricted gene flow (Artoni et al., 2006) would greatly elevate extinction risk in this population. Also, loss of genetic diversity has been reported in *Furna 2* using the D-loop mtDNA region and RAPD markers (Matoso, Artoni & Galleti Jr, 2004; Matoso et al., 2010), when compared to populations with gene flow.

## Conclusions

The causes of skeletal deformities comprise a still misunderstood and controversial issue in wild fish populations. The integration of several disciplines can generate valuable data about the nature of these malformations, mainly in non-model species, under conditions such as those seen here with *P*. aff. *fasciatus*. Some explanations of a genetic nature can be discussed as possible causes of vertebral malformations as well as the effect of inbreeding. From this perspective, only the reproduction of these *lambaris* under laboratory conditions can provide accurate information about the origin of these malformations. In light of our findings, we recommend the *Furna 2* population as a novel and highly suitable model for evo-devo studies.

## Supplemental Information

10.7717/peerj.12239/supp-1 Supplemental Information 1 Biometric parameters.

10.7717/peerj.12239/supp-2 Supplemental Information 2 Raw data: Fig. 5 and Table 2.

## Additional Information and Declarations

### Field Study Permissions

The following information was supplied relating to field study approvals (*i.e*., approving body and any reference numbers): Sampling was authorized by the Ministério do Meio Ambiente (MMA/ICMbio # 15115-1) and the Instituto Ambiental do Paraná (IAP # 15.190.528-5; Authorization 15.18).

### Data Availability

The following information was supplied regarding data availability: The radiographs are available at figshare: De la Ossa Guerra, Luz Elena (2021): Radiographs.zip. figshare. Figure. https://doi.org/10.6084/m9.figshare.16304928.v1
